# Anti-oral Microbial Flavanes from *Broussonetia papyrifera* Under the Guidance of Bioassay

**DOI:** 10.1007/s13659-019-0197-y

**Published:** 2019-01-16

**Authors:** Chang-An Geng, Meng-Hong Yan, Xue-Mei Zhang, Ji-Jun Chen

**Affiliations:** 1 State Key Laboratory of Phytochemistry and Plant Resources in West China, Kunming Institute of Botany, Chinese Academy of Sciences, 132# Lanhei Road, Kunming, 650201 Yunnan People’s Republic of China; 2 Yunnan Key Laboratory of Natural Medicinal Chemistry, Kunming, 650201 People’s Republic of China; 3 University of Chinese Academy of Sciences, Beijing, 100049 People’s Republic of China

**Keywords:** Bropapyriferol, *Broussonetia papyrifera*, Anti-oral microbial activity

## Abstract

**Abstract:**

A new flavane, bropapyriferol (**1**), and eleven known ones were isolated from the EtOAc part of *Broussonetia papyrifera* under the guidance of bioassay. The structure of compound **1** was determined by extensive 1D and 2D NMR, [*α*]_D_ spectroscopic data and quantum computation. Daphnegiravan F (**2**) and 5,7,3′,4′-tetrahydroxy-3-methoxy-8,5′-diprenylflavone (**3**) showed significantly anti-oral microbial activity against five Gram-positive strains and three Gram-negative strains in vitro. Especially, compound **3** was more potent in suppressing *Actinomyces naeslundii* and *Porphyromonas gingivalis* (MIC = 1.95 ppm) than the positive control, triclosan.

**Graphical Abstract:**

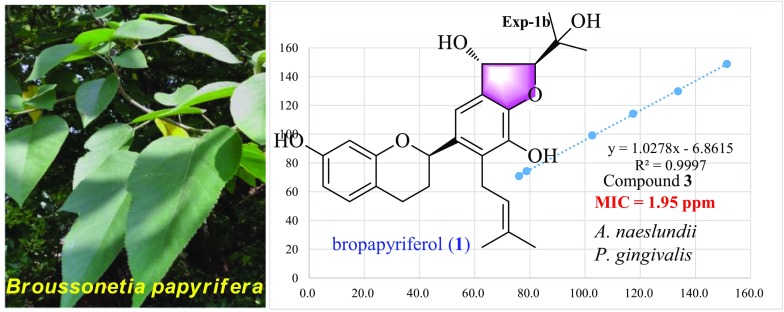

**Electronic supplementary material:**

The online version of this article (doi:10.1007/s13659-019-0197-y) contains supplementary material, which is available to authorized users.

## Introduction

*Broussonetia papyrifera*, a deciduous broadleaf plant in the family Moraceae, is native to Asia, and mainly distributed in China, Japan, Vietnam and India. *B. papyrifera* also known as paper mulberry is a highly valued plant in China, whose leaves, roots, barks and fruits are used in traditional Chinese medicines (TCMs) for diuretic, anti-rheumatic, anti-bacterial, anti-inflammatory purposes. Its bark is a good source for papermaking and leaves are high quality feed. Now, this plant widely grows in Europe, the United States and Africa as introduced a species [1, 2]. The main constituents of *B. papyrifera* are flavones, lignans, diterpenes and triterpenes [3–8]. In the previous investigation, three flavanes, kazinol B, 7,4′-dihydroxy-3′-prenylflavan and 3′-(3-methylbut-2-enyl)-3′,4′,7-trihydroxyflavane, and one triterpene, oleanolic acid, from the cortex were revealed with anti-oral microbial activity [9]. In order to clarify the anti-oral microbial effects of *B. papyrifera* and the active constituents, the preliminary bioassay revealed that the EtOAc part showed increased activity comparing to the total extraction. Thus, detailed investigation was performed on the active EtOAc part and yielded one new flavane, bropapyriferol, and eleven known ones. Herein, we report their isolation, structural elucidation and anti-oral microbial activities.

## Results and Discussion

### Structure Elucidation

Compound **1** was assigned the molecular formula of C_25_H_30_O_6_ from the positive HRESIMS at *m/z* 449.1954 ([M + Na]^+^, calcd. for 449.1940), with 11 degrees of unsaturation. The IR absorptions at 3419, 1623, 1600, 1509 and 1463 cm^−1^ were indicative for the presence of hydroxy, double bond and phenyl groups. In the ^1^H NMR spectrum, three ABX-coupling protons at *δ*_H_ 7.10 (d, *J* = 8.2 Hz, H-5), 6.87 (dd, *J* = 8.2, 2.4 Hz, H-6) and 6.93 (d, *J* = 2.4 Hz, H-8) suggested a 1,2,4-trisubstituted phenyl ring. In addition, one aromatic proton at *δ*_H_ 7.20 and four methyls at *δ*_H_ 1.75, 1.63, 1.49 and 1.41, all in singlet, were obviously recognized. In the ^13^C NMR spectrum, one methine (*δ*_C_ 75.7) and two methylenes (*δ*_C_ 30.7 and 25.8) were characteristic for a flavane skeleton when taking 12 aromatic carbons into consideration. One prenyl group was deduced from the methylene (*δ*_H_ 3.83, d, *J* = 6.6 Hz; *δ*_C_ 25.4), tri-substituted double bond (*δ*_H_ 5.49, t, *J* = 6.6 Hz; *δ*_C_ 124.8 and 130.5) and two tertiary methyls (*δ*_H_ 1.75 and 1.63, both s; *δ*_C_ 18.0 and 25.8). From the above analyses, this compound was proposed to be a prenylated flavane [6].

**Fig. 1:**
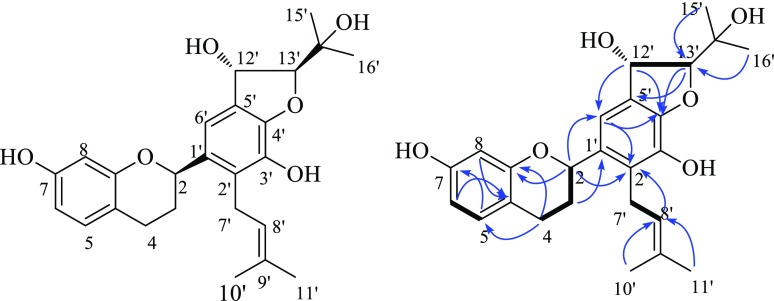
Structure of **1** and the key 2D NMR correlations (→ HMBC, ▬ ^1^H-^1^H COSY)

**Fig. 2:**
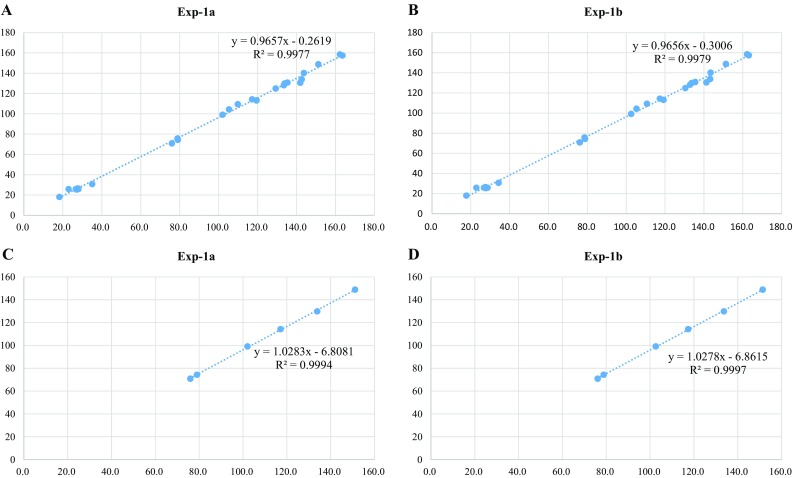
The correlations between calculated and experimental ^13^C NMR data for two possible configurations of **1** (**1a** and **1b**). All carbons (**a** and **b**) and only C-4′, 5′, 6′, 12′, 13′, 14′ (**c** and **d**) were considered

The NMR data of **1** showed similarity with those of daphnegiravan F [10], and the main difference was that compound **1** had an addition 2-hydroxypropan-2-yl group (two methyls: *δ*_H_ 1.41 and 1.49, *δ*_C_ 26.4 and 25.8; one oxygenated quaternary carbon: *δ*_C_ 70.8) and two methines (*δ*_H_ 6.07 and 4.94, *δ*_C_ 74.3 and 99.1) instead of a double bond (Table 1). Detailed analysis of the 2D NMR spectra well established its planar structure: the ^1^H-^1^H COSY correlation of H-5 with H-6, and HMBC correlations from H-6 and H-8 to C-4a, and from H-5 to C-7 verified a hydroxyl group at C-7; the HMBC correlations from H-8′ to C-2′, and from H-7′ to C-1′ and C-3′ suggested the prenyl group at C-2′; the ^1^H-^1^H COSY correlation of H-12′ with H-13′, and HMBC correlations from H-15′ and H-16′ to C-13′ indicated a hydroxyl group at C-12′ and 2-hydroxypropan-2-yl group at C-13′ (Fig. 1). The stereochemistry at C-12′ and C-13′ was deduced as *trans*-form by the coupling constant (*J*_H-12′,H-13′_ = 4.4 Hz) and comparing with that of 16-hydroxycudratrixanthone Q [11]. In order to determine the stereochemistry of **1**, the two possible configurations, 2S*,12′S*,13′S*-**1** (**1a**) and 2S*,12′R*,13′R*-**1** (**1b**), were applied to quantum computation at the B3LYP/6-311 + g(d,2p) level for the ^13^C NMR data. As shown in Fig. 2, the calculated NMR data for **1b** matched well with the experimented data, with the correlation coefficient (*R*^2^) of 0.9979, when comparing with that of the calculated **1a**. In addition, the correlation coefficient (*R*^2^) was up to 0.9997 when only the carbons (C-4′, 5′, 6′, 12′, 13′ and 14′) near C-12′ and C-13′ takes into consideration. The absolute stereochemistry was determined to be 2*R*, 12′*S*, 13′*S* by the positive [*α*]_D_ value which is consistent with the previous report and quantum calculation [12]. Thus, the structure of **1** was determined and named to be bropapyriferol.

**Table 1:** ^1^H and ^13^C NMR data of compound **1** in pyridine-*d*_5_ (*δ* in ppm, *J* in Hz)

No.	^1^H NMR (400 MHz)	^13^C NMR (100 MHz)
2	5.42, brd, 9.2	75.7, d
3	2.13, m 2.02, m	30.7, t
4	2.94, m 2.71, dd, 15.7, 2.9	25.8, t
4a	‒	113.1, s
5	7.10, d, 8.2	130.8, d
6	6.87, dd, 8.2, 2.4	109.3, d
7	‒	158.4, s
8	6.93, d, 2.4	104.3, d
8a	‒	157.4, s
1′	‒	133.8, s
2′	‒	128.1, s
3′	‒	140.1, s
4′	‒	148.8, s
5′	‒	129.8, s
6′	7.20, overlapped	114.2, d
7′	3.83, d, 6.6	25.4, t
8′	5.49, t, 6.6	124.8, d
9′	‒	130.5, s
10′	1.75, s	18.0, q
11′	1.63, s	25.8, q
12′	6.07, d, 4.4	74.3, d
13′	4.94, d, 4.4	99.1, d
14′	‒	70.8, s
15′	1.49, s	26.4, q
16′	1.41, s	25.8, q

The known compounds were determined as daphnegiravan F (**2**) [10], 5,7,3′,4′-tetrahydroxy-3-methoxy-8,5′-diprenylflavone (**3**) [13], broussoflavonol B (**4**) [14], broussonin A (**5**) [15], isoliquiritigenin (**6**) [16], uralenol (**7**) [17], broussoflavan A (**8**) [18], uralene (**9**) [19], uralenol-3-methylether (**10**) [19], abyssinone VII (**11**) [20] and 3,5,7,3′,5′-pentahydroxyflavanone (**12**) [21] by comparing with the previous reports.

### Anti-oral Microbial Activity

The total extraction and each fraction were assayed for the anti-oral microbial activity against two Gram-positive and two Gram-positive strains. As shown in Table 2, the total extraction showed activity against the four assayed strains, *A. viscosus, S. mutans, F. nucleatum* and *P. gingivalis* with MIC values of 39, 39, 39 and 19.5 ppm, respectively. After extraction by different solvents, the EtOAc part showed the highest activity with MIC values of 19.5, 19.5, 19.5 and 9.8 ppm; the *n*-butanol part exhibited slightly decreased activity with MIC values of 78.1, 39, 78.1 and 39 ppm; and the petroleum ether (PE) and water parts are inactive except for the PE part showing inhibition on *P. gingivalis* (MIC = 39 ppm).

**Table 2:** Anti-oral microbial activity of fractions from *B. papyrifera* expressed as MIC (ppm)

Fractions	*A. viscosus*	*S. mutans*	*F. nucleatum*	*P. gingivalis*
Total extraction	39	39	39	19.5
PE part	> 1250	> 1250	> 1250	39
EtOAc part	19.5	19.5	19.5	9.8
*n*-Butanol part	78.1	39	78.1	39
Water part	> 1250	> 1250	> 1250	> 1250
Triclosan^c^	3.9	3.9	7.8	7.8

Compounds **1–12** were assayed for their anti-oral microbial activity against five Gram-positive strains (*A. naeslundii*, *A. viscosus*, *S. mutans*, *S. sanguinis* and *S. sorbrinus*) and three Gram-negative strains (*A. actinomycetemcomitans*, *F. nucleatum*, *P. gingivalis*). As shown in Table 3, compound **2** exhibited significant activity against all the strains except for *S. sorbrinus*, with MIC values of 7.8, 7.8, 3.9, 15.6, 62.5, 3.9, 3.9 and 7.8 ppm, which is comparable to the positive control, triclosan. Compound **3** could obviously inhibit five Gram-positive strains and one Gram-negative strain (*P. gingivalis*), with MIC values between 1.95 and 15.6 ppm. For *A. naeslundii* and *P. gingivalis*, compound **3** showed an MIC value of 1.95 ppm, even higher than triclosan (MIC = 3.9 and 7.8 ppm, repectively). In addition, compound **10** also showed comparable inhibition with triclosan on two Gram-negative strains, *F. nucleatum* (MIC = 7.8 ppm) and *P. gingivalis* (MIC = 7.8 ppm).

**Table 3:** Anti-oral microbial activity of compounds **1–12** expressed as MIC (ppm)

Compd.	Gram-positive					Gram-negative		
	*A. na*	*A. vi*	*S. mu*	*S. sa*	*S. so*	*A. ac*	*F. nu*	*P. gi*
**1**	250	125	125	125	250	62.5	31.25	125
**2**	7.8	7.8	3.9	15.6	62.5	3.9	3.9	7.8
**3**	1.95	3.9	15.6	7.8	7.8	62.5	> 250	1.95
**4**	31.25	7.8	62.5	31.25	62.5	15.6	7.8	3.9
**5**	62.5	62.5	62.5	62.5	125	62.5	31.25	31.25
**6**	125	125	125	125	125	31.25	15.6	62.5
**7**	250	250	250	250	250	15.6	31.25	31.25
**8**	62.5	31.25	250	62.5	125	250	31.25	31.25
**9**	> 250	250	> 250	62.5	125	31.25	15.6	7.8
**10**	125	31.25	62.5	62.5	125	15.6	7.8	7.8
**11**	62.5	31.25	62.5	62.5	62.5	62.5	15.6	15.6
**12**	125	125	62.5	62.5	125	> 250	250	250
Triclosan	3.9	3.9	3.9	7.8	3.9	1.95	7.8	7.8

*A. na*, *Actinomyces naeslundii* ATCC 12104; *A. vi*, *Actinomyces viscosus* ATCC 27044; *S. mu*, *Streptococcus mutans* ATCC 25175; *S. sa*, *Streptococcus sanguinis* ATCC 10556; *S. so*, *Streptococcus sorbrinus* ATCC6715; *A. ac*, *Aggregatibacter actinomycetemcomitans* ATCC 43717; *F. nu*, *Fusobacterium nucleatum* ATCC 10953; *P. gi*: *Porphyromonas gingivalis* ATCC 33277

## Experimental

### General Procedures

Optical rotations were collected on a Jasco model 1020 polarimeter (Horiba, Tokyo, Japan). UV spectra were determined on a Shimadzu UV-2401A spectrophotometer (Shimadzu, Kyoto, Japan). IR (KBr) spectra were recorded on a Bio-Rad FTS-135 spectrometer (Bio-Rad, Hercules, California, USA). 1D and 2D NMR spectra were recorded on a Bruker AM-400 NMR or DRX-500 spectrometer with TMS as the internal standard (Bruker, Bremerhaven, Germany). MS data were collected on a VG Auto Spec-3000 spectrometer (VG, Manchester, UK) and an API Qstar Pulsar hybrid Q-TOF mass spectrometer (AB-Sciex, Framingham, MA, USA).

### Plant Materials

The plant materials of *Broussonetia papyrifera* (Linn.) L’Hér. ex Vent. were collected from Chongqing, China, in August 2009, and authenticated by Dr. Li-Gong Lei (Kunming Institute of Botany, CAS). A voucher specimen (No. 200908001) was deposited in the Laboratory of Antivirus and Natural Medicinal Chemistry, Kunming Institute of Botany, CAS.

### Extraction and Isolation

The air-dried aerial part (5 kg) of *B. papyrifera* was powdered and extracted with EtOH (90%) at room temperature for two times. The combined extract was solved in water and partitioned with petroleum ether (PE), EtOAc and *n*-butanol, successively. Based on the preliminary bioassay, the EtOAc part showed the highest anti-oral microbial activity, and was thus applied for the following investigation. The EtOAc part was separated by silica gel CC using CHCl_3_–MeOH gradient (from 9:1 to 5:5) to yield six fractions, Frs. A-F. Fr. D was fractionated by MPLC system on a MCI gel CHP20P column with MeOH-H_2_O system (5:95, 10:90, 30:70, 50:50 and 0:100) as the mobile phase to provide five fractions, Frs. D-1 to D-5. Fr. D-3 was subjected to Si CC and eluted with CHCl_3_–MeOH–H_2_O system (9:1:0.1) to generate three fractions, Fr. D-3-1 to D-3-3. Fr. D-3-2 was purified by repeated silica gel and Sephadex LH-20 CC to give compounds **5** (8 mg), **6** (11 mg) and **11** (18 mg). Fr. D-3-3 was purified by HPLC to yield compounds **7** (5 mg), **9** (7 mg), **10** (3 mg) and **12** (4 mg). Fr. D-4 was loaded on Si CC and eluted with CHCl_3_–MeOH–H_2_O system (85:15:1.5) to provide three fractions, Frs. D-4-1 to D-4-3. Compounds **2** (5 mg), **4** (3 mg) and **8** (9 mg) was obtained from Fr. D-4-1 after HPLC purification. Fr. D-4-2 was separated by HPLC to generated compounds **1** (5 mg) and **3** (6 mg).

#### Spectroscopic Data of Bropapyriferol (**1**)

Colorless gum, C_25_H_30_O_6_, [*α*]22_D_ +27.6º (*c* 0.16, CHCl_3_–MeOH, 1:1); UV (CHCl_3_–MeOH) *λ*_max_ (log *ε*) 236 (4.01) and 284 (3.69) nm; IR (KBr) *ν*_max_ 3419, 2928, 1623, 1600, 1509, 1463, 1377, 1307, 1226, 1155, 1114, 1055, 999 cm^−1^; ^1^H and ^13^C NMR data see Table 1; EIMS *m/z* 426 [M]^+^ (1), 390 (6), 350 (20), 294 (9), 227 (14), 214 (14), 185 (29), 171 (11), 123 (100); (+) HRESIMS *m/z* 449.1954 (calcd. for C_25_H_30_O_6_Na, 449.1940).

### Anti-oral Microbial Test

Microorganisms including Gram-positive [*Actinomyces naeslundii* (ATCC 12104), *Actinomyces viscosus* (ATCC 27044), *Streptococcus mutans* (ATCC 25175), *Streptococcus sanguinis* (ATCC 10556) and *Streptococcus sorbrinus* (ATCC6715)] and Gram-negative [*Aggregatibacter actinomycetemcomitans* (ATCC 43717), *Fusobacterium nucleatum* (ATCC 10953) and *Porphyromonas gingivalis* (ATCC 33277)] bacteria were used for anti-oral microbial test. All bacterial strains were obtained from the American Type Culture Collection (ATCC). A broth microdilution method was used to assess the anti-bacterial activity of the fractions and isolates by determining the minimum inhibitory concentration (MIC) [22]. In brief, a serial doubling dilution of each sample in the range 0.12–250 ppm was prepared in a 96-well plate. 100 μL of bacterial suspension was added into the 96-well plate, and the final concentration in each well was adjusted to 5 × 10^7^ CFU/mL. After incubated aerobically or anaerobically at 37 °C for about 20 h, the plate was determined by a BioTek EL × 808 Microplate Reader. The bacteria growth was indicated by the turbidity determined at OD_630_, which was performed in duplicate. The minimum inhibitory concentration (MIC) is defined as the lowest concentration of the sample at which the microorganism tested does not demonstrate visible growth. Triclosan (Purity > 99%, Ciba Specialty Chemicals) was used as the positive control.

### Quantum Computation

The ^13^C NMR and [*α*]_D_ calculations for compound **1** were carried out using Gaussian 09 program. Conformational search was achieved by Spartan ‘14 in MMFF94 s force field, and the lowest conformer was further optimized with the hf/3-21 g and DFT b3lyp/6-311 + g(d,p) methods in Gaussian 09 program package. ^13^C NMR shielding constants were calculated with the GIAO method at b3lyp/6-311 + g(d,2p) level in pyridine with PCM, which were converted into chemical shifts by referencing to TMS. [*α*]_D_ values were calculated at b3lyp/6-31 g(d,p) level based on the above DFT optimized geometries.

## Electronic supplementary material

Below is the link to the electronic supplementary material.

Electronic supplementary material 1 (PDF 292 kb)
