# Supplementary material for: Anti-oral Microbial Flavanes from Broussonetia papyrifera Under the Guidance of Bioassay
Source: Nat Prod Bioprospect. 2019 Jan 16;9(2):139–44. doi: 10.1007/s13659-019-0197-y (PMC6426910; doi:10.1007/s13659-019-0197-y)
Supplement: Supplementary file 1 — Electronic supplementary material 1 (PDF 292 kb) [file 13659_2019_197_MOESM1_ESM.pdf]

Supporting Information

Anti-oral Microbial Flavanes from *Broussonetia papyrifera* under the Guidance of Bioassay

Chang-An Geng[1,2], Meng-Hong Yan[1,2], Xue-Mei Zhang[1,2], Ji-Jun Chen[1,2,3,*]

[1] *State Key Laboratory of Phytochemistry and Plant Resources in West China, Kunming Institute of Botany, Chinese Academy of Sciences, Kunming 650201, China*

[2] *Yunnan Key Laboratory of Natural Medicinal Chemistry, Kunming 650201, China*

[3] *University of Chinese Academy of Sciences, Beijing 100049, China*

[*] *Corresponding Author: Ji-Jun Chen, State Key Laboratory of Phytochemistry and Plant Resources in West China, Kunming Institute of Botany, Chinese Academy of Sciences, 132# Lanhei Road, Kunming 650201, Yunnan, China. Tel: +86-871-65223265. Fax: +86-871-65227197. E-mail: chenjj@mail.kib.ac.cn (J.J. Chen).*

Computational details

Conformational search was achieved by Spartan '14 in MMFF94s force field, and the lowest conformer was further optimized with the hf/3-21g, b3lyp/6-31g(d,p) and DFT b3lyp/6-311+g(d,p) methods in Gaussian 09 program package. ^{13}C NMR shielding constants were calculated with the GIAO method at b3lyp/6-311+g(d,2p) level in pyridine with PCM, which were converted into chemical shifts by referencing to TMS. $[\alpha]_D$ values were calculated at b3lyp/6-31g(d,p) level based on the above DFT optimized geometries.[1]

Reference

1. Frisch, M. J.; Trucks, G. W.; Schlegel, H. B.; Scuseria, G. E.; Robb, M. A.; Cheeseman, J. R.; Scalmani, G.; Barone, V.; Mennucci, B.; Petersson, G. A.; Nakatsuji, H.; Caricato, M.; Li, X.; Hratchian, H. P.; Izmaylov, A. F.; Bloino, J.; Zheng, G.; Sonnenberg, J. L.; Hada, M.; Ehara, M.; Toyota, K.; Fukuda, R.; Hasegawa, J.; Ishida, M.; Nakajima, T.; Honda, Y.; Kitao, O.; Nakai, H.; Vreven, T.; Montgomery, J. A.; Peralta, Jr. J. E.; Ogliaro, F.; Bearpark, M.; Heyd, J. J.; Brothers, E.; Kudin, K. N.; Staroverov, V.N.; Keith, T.; Kobayashi, R.; Normand, J.; Raghavachari, K.; Rendell, A.; Burant, J.C.; Iyengar, S. S.; Tomasi, J.; Cossi, M.; Rega, N.; Millam, J. M.; Klene, M.; Knox, J. E.; Cross, J. B.; Bakken, V.; Adamo, C.; Jaramillo, J.; Gomperts, R.; Stratmann, R. E.; Yazyev, O.; Austin, A. J.; Cammi, R.; Pomelli, C.; Ochterski, J. W.; Martin, R. L.; Morokuma, K.; Zakrzewski, V. G.; Voth, G. A.; Salvador, P.; Dannenberg, J. J.; Dapprich, S.; Daniels, A. D.; Farkas, O.; Foresman, J. B.; Ortiz, J. V.; Cioslowski, J.; Fox, D. J. Gaussian 09, Revision C.01; Gaussian, Inc., Wallingford CT: 2010.

Standard orientation of **1a**

Center Number	Atomic Number	Atomic Type	Coordinates (Angstroms) X	Y	Z
1	6	0	-4.910928	-3.252546	-0.724752
2	6	0	-5.821186	-2.633203	0.135449
3	6	0	-5.428430	-1.488664	0.815607
4	6	0	-4.154872	-0.927375	0.669642
5	6	0	-3.266006	-1.565733	-0.204109
6	6	0	-3.637463	-2.723154	-0.897082
7	6	0	-3.725321	0.308536	1.429152
8	6	0	-2.200168	0.445217	1.430355
9	6	0	-1.655193	0.208581	0.017177
10	8	0	-1.986650	-1.129540	-0.423610
11	6	0	-0.151245	0.357204	-0.077735
12	6	0	0.437234	1.534934	-0.591167
13	6	0	1.836931	1.646420	-0.592252
14	6	0	2.595261	0.587796	-0.112124
15	6	0	2.024532	-0.566032	0.384049
16	6	0	0.644024	-0.694063	0.398763
17	6	0	-0.366007	2.701205	-1.147487
18	6	0	-0.880543	3.637275	-0.076448
19	6	0	-2.075177	4.238920	-0.008039
20	6	0	-2.402549	5.173955	1.131286
21	6	0	-3.179327	4.084052	-1.023590
22	8	0	-5.328707	-4.382776	-1.378819
23	8	0	2.438460	2.781336	-1.073417
24	8	0	3.967279	0.590922	-0.109403
25	6	0	4.405566	-0.618036	0.594753
26	6	0	3.140870	-1.493791	0.801204
27	8	0	3.124998	-1.887853	2.178264
28	6	0	5.553503	-1.253858	-0.210220
29	6	0	6.039869	-2.532581	0.471204
30	6	0	6.709674	-0.256751	-0.372116
31	8	0	5.060979	-1.651359	-1.499015
32	1	0	-6.809366	-3.057208	0.260567
33	1	0	-6.130986	-1.012616	1.493072
34	1	0	-2.916562	-3.183854	-1.564683
35	1	0	-4.169795	1.203097	0.974608
36	1	0	-4.102281	0.266333	2.455711
37	1	0	-1.746419	-0.289229	2.103992
38	1	0	-1.894571	1.437227	1.768934
39	1	0	-2.149355	0.903504	-0.668030
40	1	0	0.165888	-1.598063	0.757101
41	1	0	0.289112	3.264898	-1.818210
42	1	0	-1.182198	2.324345	-1.766249
43	1	0	-0.160870	3.854523	0.710558
44	1	0	-3.287854	4.830760	1.680138
45	1	0	-1.574245	5.258274	1.837356
46	1	0	-2.636914	6.178330	0.759011
47	1	0	-3.398540	5.048995	-1.495914
48	1	0	-2.944429	3.376648	-1.818899
49	1	0	-4.108322	3.758812	-0.540832
50	1	0	-4.617464	-4.716060	-1.935509
51	1	0	3.392957	2.689230	-0.965459
52	1	0	4.761779	-0.308707	1.581526
53	1	0	3.173044	-2.381847	0.160547
54	1	0	2.373929	-2.472077	2.324645
55	1	0	6.411891	-2.322031	1.476288
56	1	0	5.241397	-3.271776	0.547921
57	1	0	6.849368	-2.966882	-0.118851
58	1	0	6.378622	0.648305	-0.886019
59	1	0	7.507962	-0.717172	-0.957865
60	1	0	7.116235	0.036145	0.600015
61	1	0	4.758799	-0.857599	-1.956589

Standard orientation of **1b**

Center Number	Atomic Number	Atomic Type	Coordinates (Angstroms) X	Y	Z
1	6	0	-5.222203	-2.951287	-0.498605
2	6	0	-5.972597	-2.313034	0.492273
3	6	0	-5.415859	-1.233416	1.163955
4	6	0	-4.130632	-0.755593	0.884245
5	6	0	-3.404098	-1.411244	-0.117383
6	6	0	-3.942541	-2.504082	-0.805501
7	6	0	-3.522476	0.410115	1.632191
8	6	0	-2.002271	0.442303	1.450137
9	6	0	-1.647976	0.232293	-0.026015
10	8	0	-2.130446	-1.056185	-0.474857
11	6	0	-0.157735	0.276524	-0.291660
12	6	0	0.458085	1.429289	-0.829242
13	6	0	1.854743	1.444311	-0.974757
14	6	0	2.581276	0.321163	-0.604029
15	6	0	1.979617	-0.819464	-0.114084
16	6	0	0.604228	-0.846176	0.059747
17	6	0	-0.311215	2.667975	-1.263662
18	6	0	-0.615628	3.613807	-0.123131
19	6	0	-1.747206	4.290718	0.112027
20	6	0	-1.856233	5.222548	1.294942
21	6	0	-2.985080	4.227666	-0.747062
22	8	0	-5.799996	-4.015548	-1.141245
23	8	0	2.483639	2.553125	-1.481007
24	8	0	3.948198	0.250317	-0.693405
25	6	0	4.338271	-1.125282	-0.367031
26	6	0	3.061714	-1.837135	0.156326
27	8	0	2.938538	-3.064005	-0.572676
28	6	0	5.531805	-1.070260	0.603961
29	6	0	6.696208	-0.288171	-0.019533
30	6	0	5.976087	-2.482760	0.982917
31	8	0	5.114547	-0.441113	1.825794
32	1	0	-6.967739	-2.672411	0.721312
33	1	0	-5.994289	-0.743506	1.941538
34	1	0	-3.343785	-2.979784	-1.575452
35	1	0	-3.952430	1.353444	1.272144
36	1	0	-3.774630	0.344625	2.695133
37	1	0	-1.526135	-0.349630	2.037592
38	1	0	-1.586720	1.395664	1.782658
39	1	0	-2.163671	0.992661	-0.619323
40	1	0	0.101775	-1.726709	0.442591
41	1	0	0.302603	3.196471	-1.999251
42	1	0	-1.222694	2.372490	-1.785362
43	1	0	0.215105	3.767550	0.562950
44	1	0	-2.071544	6.246953	0.968460
45	1	0	-2.680695	4.926255	1.954681
46	1	0	-0.937218	5.240680	1.884046
47	1	0	-3.862118	3.961498	-0.145636
48	1	0	-3.196066	5.211779	-1.182037
49	1	0	-2.908733	3.516050	-1.568883
50	1	0	-5.189495	-4.368005	-1.796940
51	1	0	3.421198	2.350170	-1.586286
52	1	0	4.632331	-1.609078	-1.302501
53	1	0	3.145125	-2.048043	1.227844
54	1	0	2.139584	-3.517341	-0.284117
55	1	0	6.397813	0.734348	-0.261087
56	1	0	7.528085	-0.250292	0.686841
57	1	0	7.043442	-0.765614	-0.940164
58	1	0	5.171643	-3.038197	1.467015
59	1	0	6.813477	-2.419520	1.680613
60	1	0	6.296184	-3.040567	0.100101
61	1	0	4.844037	0.460508	1.614515

Experimental ^{13}C NMR data of **1** and calculated ^{13}C NMR NMR data of **1a** and **1b**

No.	cal-1a	cal-1b	Exp
**2**	78.9	78.6	75.7
**3**	35.1	34.3	30.7
**4**	28.0	28.7	25.8
**4a**	119.5	119.2	113.1
**5**	135.3	135.6	130.8
**6**	109.9	110.7	109.3
**7**	162.2	162.3	158.4
**8**	105.3	105.3	104.3
**8a**	163.5	163.1	157.4
**1'**	142.6	143.2	133.8
**2'**	133.4	132.9	128.1
**3'**	143.8	143.5	140.1
**4'**	151.1	151.4	148.8
**5'**	133.9	133.6	129.8
**6'**	117.2	117.4	114.2
**7'**	27.1	27.8	25.4
**8'**	129.3	130.4	124.8
**9'**	141.9	141.3	130.5
**10'**	18.2	17.7	18
**11'**	26.6	26.8	25.8
**12'**	79.0	78.8	74.3
**13'**	102.0	102.6	99.1
**14'**	76.0	76.1	70.8
**15'**	27.7	27.6	26.4
**16'**	23.0	22.9	25.8

Calculated $[\alpha]_D$ values for **1b**

Compounds	chloroform	methanol
2R,12'S,13'S-1b	+167.76	+109.95
2S,12'R,13'R-1b	–167.76	–109.95
